# Long- and Short-Range Electrostatic Fields in GFP Mutants: Implications for Spectral Tuning

**DOI:** 10.1038/srep13223

**Published:** 2015-08-19

**Authors:** M. Drobizhev, P. R. Callis, R. Nifosì, G. Wicks, C. Stoltzfus, L. Barnett, T. E. Hughes, P. Sullivan, A. Rebane

**Affiliations:** 1 Department of Physics, Montana State University, Bozeman MT 59717; 2 Department of Chemistry and Biochemistry, Montana State University, Bozeman MT 59717; 3 NEST, Istituto Nanoscienze—CNR and Scuola Normale Superiore, I-56127 Pisa, Italy; 4 Department of Cell Biology and Neuroscience, Montana State University, Bozeman MT 59717; 5 National Insitute of Chemical Physics and Biophysics, Tallinn 12618, Estonia

## Abstract

The majority of protein functions are governed by their internal local electrostatics. Quantitative information about these interactions can shed light on how proteins work and allow for improving/altering their performance. Green fluorescent protein (GFP) and its mutation variants provide unique optical windows for interrogation of internal electric fields, thanks to the intrinsic fluorophore group formed inside them. Here we use an all-optical method, based on the independent measurements of transition frequency and one- and two-photon absorption cross sections in a number of GFP mutants to evaluate these internal electric fields. Two physical models based on the quadratic Stark effect, either with or without taking into account structural (bond-length) changes of the chromophore in varying field, allow us to separately evaluate the long-range and the total effective (short- and long-range) fields. Both types of the field quantitatively agree with the results of independent molecular dynamic simulations, justifying our method of measurement.

Electric fields inside proteins play a central role in enzymatic function^1^,^2^, firing action potentials in neurons^3^,^4^, color vision^5^, etc. In GFP and its mutants, a variety of spectral hues is usually attributed to different electrostatic environments of their chromophore. By mutating the local protein surrounding, while keeping the structure of the chromophore unchanged, one can tune the optical transition frequency within an impressive spectral range of ~65 nm^6^,^7^, see Fig. 1. Structural analysis^7^ and quantum simulations^8^,^9^,^10^,^11^ suggest that the main part of this tuning is due to variations of electrostatic fields around the chromophore. Therefore, on one hand, one can use GFP and its mutants as model systems to investigate internal electric fields, especially because the structure of many of them is known, and on the other hand, this knowledge could lead to the creation of new fluorescent proteins (FPs) with improved optical properties.

Classical Stark shift methods measure internal fields in proteins by using either UV-vis (electronic) or infrared (vibrational) absorption spectroscopy^2^,^12^,^13^. The development of alternative approaches, based on a combination of one- and two-photon excited fluorescence, would allow evaluating these fields in biologically intact living cells and tissues and in the high-resolution fluorescence microscopy conditions.

The one-photon absorption (1PA) spectrum of a chromophore in a weak uniform electric field ***E***experiences a spectral shift Δ*ν* (Δ*ν* = *ν* – *ν*_0_, where *ν* and *ν*_0_ are the transition frequencies in protein and in vacuum, respectively), described by a linear Stark formula: *h*Δ*ν* = −Δ***μ*** • ***E***, where Δ***μ*** is the difference between the permanent electric dipole moments in the excited (*e*) and ground (*g*) states, Δ***μ*** = ***μ***_e _− ***μ***_g_. The above relation between the field and spectral shift could in principle be used for estimation of the internal field ***E*** inside a protein by measuring the shifts of absorption peaks, if Δ***μ*** and *ν*_0_ were known. We have previously demonstrated that in a series of red FPs the value of Δ***μ*** can be obtained from the two-photon absorption (2PA) cross section, measured at the red side of the S_0_–S_1_ transition band^14^. Our results have shown however that Δ***μ*** significantly changes from one protein to another (for the series with the same chromophore) and therefore the higher order, quadratic Stark effect should be considered. This can be realized by admitting that because of the presence of quite strong local fields inside proteins (~10^7^ V/cm); Δ***μ*** itself becomes a function of ***E*** due to the induced (through polarizability) dipole moments. A simple analysis of the quadratic Stark effect in a series of red FPs made it possible to obtain a crude, order-of-magnitude estimation of the fields^14^.

However, our initial simplified model based on quadratic Stark shifts did not take into account some important details. First, it considered the chromophore to be one-dimensional, which probably is not very accurate for the red FPs^15^. Second, the field was treated as uniform, i.e. not changing across the π-conjugated system of the chromophore. Although potentially justifiable for the long-range Coulomb interactions, this approximation does not always work for the local short-range interactions (e.g. hydrogen bonding (HB)). Including such interactions is critical, because they can significantly change the values of Δ***μ***^16^ and transition energy^16^,^17^,^18^. Finally, only pure electronic effects were assumed, i.e. the change of atom positions upon applying the field was disregarded. It is known, however, that structural changes, namely the changes of the bond-length alternation (BLA) inside a chromophore, can result in significant shifts of the optical transition energy and corresponding changes of Δ***μ*** values^17^,^18^,^19^.

Here we study a set of 26 GFP mutants, all containing the same chromophore in the anionic state, as well as the solution of a model synthetic chromophore, p-hydroxybenzylidene-2,3-dimethylimidazolinone anion (HBDI^−^) in alkaline D_2_O. Strictly speaking, mutants/homologs in this series differ for the first chromophore –forming amino acid (Ser65 in the original *av*GFP). However, this amino acid’s side chain does not participate to the π-conjugated system, so that the chromophore structure can be considered the same as far as its electronic properties are concerned. We selected the GFP series because their chromophore has simpler, quasi one-dimensional structure, compared to the red FP chromophore. As a first approximation, we consider a simple model, where only pure electronic effects were taken into account (i.e. the positions of atoms are fixed) and the total (short- and long-range) field is considered. To further elucidate the role of the local chromophore environment, we then refined the model by separating the long-range Coulomb interactions from the short-range (hydrogen bonding) specific interactions. This leads us to an expanded “effective” chromophore or, in other words, cluster, which in addition to the chromophore itself involves a number of amino acid residues and water molecules making hydrogen bonds with it. This cluster is embedded in the electrostatic surrounding created by the rest of the protein and water molecules. This latter approach made it possible to evaluate the long-range quasi-uniform fields. Independent evaluations of the total and the long-range fields complement each other and allow us to estimate the local, short-range interactions. Both total and long-range fields measurements are verified by molecular dynamics (MD) simulations.

## Results and Discussion

### Pure electronic Stark effect model. Total effective electric fields

Here we assume a one-dimensional chromophore, interacting through its electronic system with the electric field of protein surrounding. We suppose that the chromophore structure, i.e. its bond lengths and angles do not change upon transition from one local surrounding to another. The difference of the chromophore dipole moments Δ***μ*** will contain a field-independent (vacuum) part Δ***μ***_0_ and a field-induced part, Δ***μ***_ind_. In the point dipole approximation, we can write Δ*μ*_ind_ = Δ*αE*_Δ*μ*_, where *E*_Δ*μ*_ is the projection of the electric field on the direction of the Δ***μ***_0_ vector and Δ*α* is the change of the chromophore polarizability upon excitation. For simplicity, we will call *E*_Δ*μ*_ the field in what follows. In our model we assume that Δ*α* does not depend on the field (no hyperpolarizability effects). We define a positive molecular *x*-axis along the direction from the center of the imidazolinone ring to the center of the phenol ring which virtually coincides with the direction of the Δ***μ***_0_ vector^20^,^21^,^22^,^23^,^24^,^25^. The total change of the permanent dipole moment (projection onto x-axis) then reads^26^:


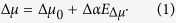


(In our previous paper^14^ the coefficient ½ was used in the second term of eq. (1) following an erroneous presentation in some previous literature, see e.g. Ref. 26 A correct consideration^27^ should not contain it.)Equation (1) can be solved for the field to obtain:


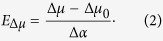


This equation establishes a linear correlation between the experimentally measurable permanent dipole difference and the electric field. Since the field can be non-uniform across the length of the chromophore, we call it effective field, i.e. producing the same effect as the uniform field of magnitude *E*_Δ*μ*_. This field also shifts the electronic transition frequency relative to that in vacuum via the second-order Stark effect, as follows^27^:


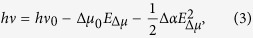


where *h* is the Planck’s constant. Substituting the expression for *E*_Δ*μ*_ (2) into (3) and expressing frequencies in wavenumbers (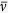 = *ν*/*c*, where *c* is the speed of light), we obtain the relation between the experimentally measurable parameters 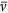 and Δ*μ*:


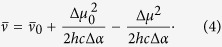


This equation shows that the transition frequency as a function of Δ*μ* is described by a parabola with the extremum found at Δ*μ* = 0. Its curvature is defined by −(2*hc*Δ*α*)^−1^.

Figure 2 presents the experimental dependence of the Boltzmann-broadened 0–0 transition frequency on Δ*μ*, for the anionic GFP chromophore in a variety of local environments (see Methods for the measurement details). Note that only the 0–0 2PA transition can be safely expected to reflect the dipole change, due to vibronic coupling effect^28^.

Fitting of all experimental data to the function 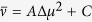 (full black line in Fig. 2) provides the coefficients *A* = −(2*hc*Δ*α*)^−1^ = 72 ± 4 cm^−1^D^−2^ and 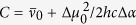 = 19300 ± 70 cm^−1^. From the first coefficient, we obtain Δ*α* = −35 ± 2 Å^3^. Substituting this value, as well as the transition frequency measured in the gas phase, 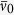 = 20725 cm^−1^, Ref. 29, into the expression for *C*, we obtain Δ*μ*_0 _= 4.47 ± 0.16 D. Note that both Δ*μ*_0_ and Δ*α* represent some effective values averaged over several different structural variants of the chromophore, e.g. including different bond-length patterns in different classes of proteins or different twisting conformations in water *vs*. protein environment. Despite this, the experimental values of Δ*μ*_0_ and Δ*α* fall in the range predicted by quantum calculations for the HBDI^−^ chromophore in vacuum (see Table 1 and Supplementary Tables S1 and S2).

By knowing the values of the parameters Δ*α* and Δ*μ*_0_, we find the fields *E*_Δ*μ*_ from (2) for all the systems studied, see Supplementary Table S3. For some of the proteins, crystallographic structures are available and the corresponding fields can therefore be evaluated theoretically using MD simulations and these results can then be compared to experimental values. Note that the expected outcomes rely only on molecular mechanics (MM) treatment of the protein around the chromophore. In particular, the partial charges in the force field used (see Methods) are obtained by fitting the electrostatic potential (calculated at the Hartree-Fock level) on molecular surfaces and therefore this approach is expected to be sufficiently accurate. For such a comparison we have selected four proteins with very different (in magnitude and direction) electric fields, including YFP-variant citrine (pdb files: 3DPW and 1HUY), EGFP (pdb: 4EUL and 2Y0G), mTFP1.0 (pdb: 2HQK), and mTFP0.7 (pdb: 2OTB).

Figure 3(a) shows the changes of electrostatic potential as a function of distance along the straight line connecting the atoms CA2 and CE2. The potentials at the CE2, CD2, CG2, CB2, and CA2 atoms, closest to the line are shown versus the cumulative distance between the projections of the atoms on that line. Selection of these atoms is justified by the theoretical calculations^25^,^30^,^31^ demonstrating that the most significant change of electronic density upon the S_0_ → S_1_ excitation occurs at the CG2, CB2, CA2, CE2, CD2, C2, CE1, CD1, and OH atoms, and that the vector connecting the CA2 and CE2 atoms is virtually co-directed with Δ***μ***_0_. The three central atoms, CG2, CB2, and CA2, making the bridge between the phenolate and imidazolinone rings are known to be the main players in the charge-transfer process^32^,^33^,^34^. The electric field projected onto the CA2-CE2 direction should therefore be responsible for the induced part of Δ***μ*** and for the shifts of transition energy. To obtain *E*_Δ*μ*_, we took a negative gradient of the calculated electrostatic potential function (i.e. the slopes of the straight lines in Fig. 3(a)). The correlation between the experimentally measured and simulated fields for four proteins is shown in Fig. 3(b). Note that both the magnitude and the sign of the field are reproduced in MD surprisingly well, especially for mTFP0.7, mTFP1.0, and EGFP. The experimental values vary in a wide range, from −8.6 MV/cm in mTFP0.7 to +7.4 MV/cm in EGFP, reaching +25.4 MV/cm in citrine, which represent typical order of magnitude in proteins^12^,^13^,^35^,^36^,^37^. The wide range of the fields observed is due to significant changes of local interactions. In particular, the increase of the component of the field directed from phenolate to imidazolinone (negative *x*) by ~16 MV/cm when going from EGFP to mTFP0.7 (Fig. 3(b)) can be qualitatively explained by the presence of the unique, positively charged H197 and negatively charged E148 residues near the chromophore in mTFP0.7 7,18.

Our simple Stark effect model, Fig. 2, also explains the large red shift of yellow FP citrine *vs*. EGFP by the much smaller Δ*μ* value of the former, i.e. 1.5 *vs*. 3.6 D. The intermediate red shifts in T203I mutants (orange pentagons in Fig. 2) are also due to a decrease of Δ*μ*. Structurally, in citrine, the key mutation which significantly red-shifts the absorption is T203Y^38^. This substitution reduces the number of hydrogen bonds at the phenolate oxygen from 3 to 2 and, also, creates a new, π-π stacking interaction of the Tyr203 phenol group with the chromophore^38^. It is interesting that the main part of the red shift is due to the loss of a hydrogen bond because even aliphatic substitutions at the same position, i.e. T203V and T203I produce a similar, although slightly smaller, red shift (see Ref. 39 and Fig. 2). It has been proposed^39^ that the extra hydrogen bond, created by the threonine hydroxyl in EGFP, pulls the electron density to the phenolate oxygen, thus stabilizing the ground state and shifting the transition energy up as compared to the EGFP T203I mutant. This interaction is strongly localized near the OH atom and appears to be not fully detectable by averaging the potential changes over the further removed atoms CE2, CD2, CG2, CB2, and CA2, as is done in Fig. 3(a). On the other hand, it significantly changes Δ*μ* of the chromophore and should therefore be observed as a contribution to the effective experimental field. In fact, the difference between the experimental and simulated fields in citrine ~10 MV/cm, see Fig. 3(b), is probably due to this unaccounted short-range interaction. In the next section we refine the model to separate the effects of the short- and long-range fields.

### Refined model that includes structural changes of the chromophore. Long-range electric fields

In an attempt to refine the model and elucidate the role of the short-range interactions, which are probably the main cause of discrepancies between the effective experimental and simulated field values (cf. citrine), we now consider a cluster, consisting of the π-conjugated system of the chromophore itself and its closest surrounding. Within the cluster, the chromophore interacts specifically with the inner shell of several chemical groups. In particular, we consider the hydrogen bonding to the OH and O2 atoms of the chromophore as a main source of the short-range interactions. These hydrogen-bonded groups can perturb the initial (vacuum) dipole moments ***μ***_g_ and ***μ***_e_. As a result, the dipole moment difference of the chromophore in the hydrogen-bonded cluster, Δ*μ*_HB_, can differ from that observed in vacuum. We will further assume that the Δ*μ*_HB_ is invariant for a series of FP mutants with the same number and character of hydrogen bonds in the cluster.

To obtain the total dipole moment difference, Δ*μ*, we should include, in addition to Δ*μ*_HB_, a contribution that is induced by the long-range electric field, *E’*_Δ*μ*_, created by the outer parts of the protein. We use the prime sign to distinguish the long-range part of the field from the total field (*E*_Δ*μ*_). Using the same approach, as in (1)–(2), and again assuming that Δ*α* does not depend on the long- and short-range field variations, we now obtain for the long-range field within a class of proteins with particular Δ*μ*_HB_:


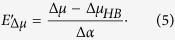


The unknown parameter Δ*μ*_*HB*_ can be obtained either from quantum mechanical calculations or from comparison of experimental data with the refined model, describing optical transitions in a series of proteins with the same local surrounding.

#### Quantum mechanical calculations of Δμ_HB_ and Δ*α*

For generality, we performed quantum mechanical calculations of the dipole moment differences in vacuum and in four different hydrogen-bonding clusters by using the same method (see Methods for details).

First, the chromophore with five hydrogen-bonded water molecules represented the cluster encountered in water solution (cluster (1)). The cluster (2) included the chromophore with five hydrogen-bonding contacts (5-HB cluster), corresponding to mTFP0.7 structure. It contained His163 (modeled with neutral imidazole group), Ser146 (modeled with the CH_3_OH group), and an H_2_O molecule, all three bonded to the phenolate oxygen, as well as Arg95 (modeled with positively charged guanidinium group) and another H_2_O molecule, both bound to the imidazolinone oxygen. The next cluster (3) corresponded to the EGFP where His148, Thr203, and a water molecule were H-bonded to the phenolate oxygen and Arg96 and Gln94 were H-bonded to the imidazolinone oxygen. The cluster (4), corresponding to citrine, contained the chromophore with His148 and H_2_O both H-bonded to the phenolate oxygen and R96 (H-bonded to imidazolinone oxygen) and Q94 (probably weakly or not H-bonded, but very close to imidazolinone oxygen). The Q94 is retained in the citrine cluster for easier comparison with EGFP.

We select the molecular frame, such that the *x*-axis is directed from the geometrical center of the imidazolinone ring to the geometrical center of the phenol ring that is virtually parallel to the (calculated) transition dipole vector. The *y*-axis is directed orthogonally and lies in the average molecular plane, as shown in the Fig. 4. The *z*-axis makes a right triad with *x* and *y*. The results of the calculations are presented in Table 1.

The calculation shows that the Δ***μ***_*HB*_ vector is directed almost perfectly along the *x*-axis, similarly to what was found in previous calculations for the HBDI^−^ chromophore in vacuum, water, and GFP protein (Supplementary Table S1), thus justifying the one-dimensional model of the chromophore.

Note that the calculated value of Δ*μ* in vacuum (4.35 D) is in good agreement with the experiment (4.47 ± 0.16 D). The addition of five hydrogen-bonded water molecules to the chromophore slightly increases the Δ*μ* magnitude (~5.2 D). On the other hand, the Δ*μ* value decreases (compared to vacuum) in the mTFP-derived 5-HB cluster to 3.3 D (entry 3) which, according to our analysis, can be mainly attributed to the positively-charged Arg95. Interestingly, removing all the surrounding groups, but keeping the chromophore structure the same as it was found after optimization in the cluster, produces only minor change of the Δ*μ*, i.e. from 3.3 to 2.9 D, cf. entries 3 and 4 of Table 1. This important result demonstrates that in this case the change of Δ*μ* in cluster *vs.* vacuum is primarily due to the change of the chromophore structure, namely to a different bond-length alternation pattern (cf. Ref. 17), and that the electronic redistribution is coupled to the structural change. Another interesting result is that in the citrine cluster, where there are only three hydrogen bonds (entry No. 6), the Δ*μ*_*HB*_ value is further significantly reduced compared to that observed in the 5-HB clusters.

To obtain the Δ*α* value, the dependence of Δ*μ* on *E*_Δ*μ*_ was calculated by applying a uniform electric field along the *x*-axis of the chromophore (Methods and Supplementary Fig. S1). The resulting value, Δ*α* = −49 Å^3^, is in reasonable agreement with the experimental one (−35 Å^3^). Using the calculated parameters of Δ*α* and Δ*μ*_*HB*_ (Table 1), as well as experimental values of Δ*μ*, we evaluated the long-range field *E′*_Δ*μ*_ by using eq. (5). For four selected proteins the results are presented in Fig. 3d (abscissa, green stars) and for the rest of the proteins – in Supplementary Table S3.

#### Experimental evaluation of Δμ_HB_

We also carried out an independent evaluation of the parameter Δ*μ*_*HB*_ for the subset of 5-HB proteins using experimental measurements. To this end, we considered the dependence of transition frequency on the electric field by separating the effects of structural and pure electronic responses. First, we assume that both short- and long-range electric fields perturb molecular structure such that the alternation of the single and double bond lengths changes accordingly. To obtain the dependence of transition frequency on the field and hence on Δ*μ*, the resonance theory was applied in the simple two-forms two-states (2F2S) implementation^40^,^41^,^42^ adapted to the GFP chromophore in^17^,^18^,^19^,^28^,^43^. This model predicts the quadratic dependence of the optical transition frequency on the value of Δμ, Ref. 19. We then include the pure electronic interaction of the chromophore system with the long-range electrostatic field through the second-order Stark effect. As a result, the following dependence of the transition frequency on Δ*μ* was obtained (see Supplementary Discussion):


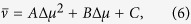


with


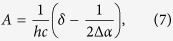



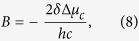



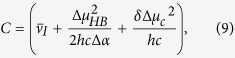


where 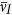 is the parameter describing the coupling between the two resonating forms^19^, 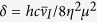, *η* is a dimensionless parameter whose physical meaning consists in the “screening” of the charge-transfer transition via redistribution of those electrons clouds which are not directly involved in the electron transfer related to Δ*μ*^19^,^44^, *μ* is the transition dipole moment between the ground and excited states, Δ*μ*_c_ is a constant, corresponding to the difference between the dipole moments in the excited and ground states in a particular chromophore geometry corresponding to the effective bond length alternation equal to 0 (see Supplementary Fig. S2). The three coefficients, *A*, *B*, and *C* were found from the best fit of the 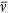 vs Δ*μ* dependence for a subset of proteins with 5 HBs in the cluster to a second order polynomial (see Supplementary Fig. S3). Since there are four unknown parameters (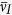, *δ*, Δ*μ*_*c*_, and ΔΔ*μ*_*HB*_) in the system of three equations (7)–(9), 13, 14, we use an additional equation which is obtained by considering the 2F2S model in the zero field (vacuum), i.e. *E’*_Δ*μ*_ = 0 and Δ*μ* = Δ*μ*_0_. This equation reads (see Supplementary Discussion):


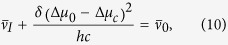


where 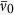 = 20725 cm^−1^ is the transition frequency in vacuum^29^. The resulting experimental parameters involved in the model were obtained by solving eqs. (7)–(10), 13, 14, 19 (See Supplementary Discussion) and are presented in Table 2. Most of them were also independently calculated quantum-mechanically (Table 2) and agree well with the experiment.

Combining the values of Δ*μ*_*HB *_= 3.4 D and Δ*α* = −35 Å^3^, both found experimentally, the fields *E ′*_Δ*μ*_ were calculated according to (5) (Supplementary Table S3).

#### Comparison of the long-range fields, obtained from Δμ and from MD simulations

To check these two sets of data (with Δ*μ*_*HB*_ and Δ*α* either calculated or found from experimental measurements) against the MD simulations, the same group of mutants was selected as in Fig. 3(a)–(b). Now the charges on the chromophore and 5 amino acids/water molecules (4 in case of citrine) around it were disregarded in calculating the potentials. Figure 3(c) shows the variation of potentials along the straight line connecting the CE2-CD2-CG2-CB2-CA2 chain of atoms, which provided the fields for all the proteins.

Figure 3(d) compares the MD simulated fields (*y*-axis) with the fields obtained from model eq. (5). The correlation between the MD simulations and the model is good (particularly for the calculated Δ*μ*_*HB*_ and Δ*α*, green stars). For citrine it is improved significantly, as compared to the case of the total effective field, cf. Figure 3(b). The long-range electric fields inside the proteins under study vary in the range from ~1.6 MV/cm in citrine to −13 MV/cm in mTFP 0.7. This variation is smaller than that of the total effective field, Fig. 3(b), which emphasizes the importance of the short-range interactions in the wide spectral tuning of the FP. The direction of the long-range field, found in the four mutants under study is opposite to that of Δ*μ*, i.e. *E ′*_Δ*μ*_ is directed from the phenolate to the imidazolinone. Note that in the 5-HB proteins, mTFP0.7, mTFP1.0, and EGFP, the field was shifted to more negative values by ~9 MV/cm, when the cluster was discarded (cf. Figure 3 (b,d), blue stars). This is because the positively charged R96 residue (EGFP notation), being a part of the cluster in the three proteins, created the short-range field in the positive direction, i.e. from imidazolinone to phenolate, see Fig. 4.

In the case of citrine, the long-range field value is much better reproduced by MD simulations and it is also much closer to that of EGFP, as compared to what was observed for the total effective field, cf. Figure 3(d,b). This reflects the fact that the large part of structural changes between the two mutants occur at a short range. In particular, the reduced number of hydrogen bonds in citrine vs. EGFP results in a significant decrease of Δ*μ*, from 3.6 D (EGFP) to 1.5 D (citrine), which can be simulated by a large increase of the short-range contribution to the effective field. Note that the much lower value of Δ*μ* in citrine is consistent with the previous observation of the smaller static first hyperbolarizability in EYFP compared to EGFP^45^,^46^. A little upward shift of the long-range field, by ~5 MV/cm, in citrine *vs.* EGFP, cf. green stars in Fig. 3(d), can be tentatively attributed to the dipole-induced contribution of the Tyr203 phenol group.

### Field-induced broadening of the absorption spectrum

**Figure 1:**
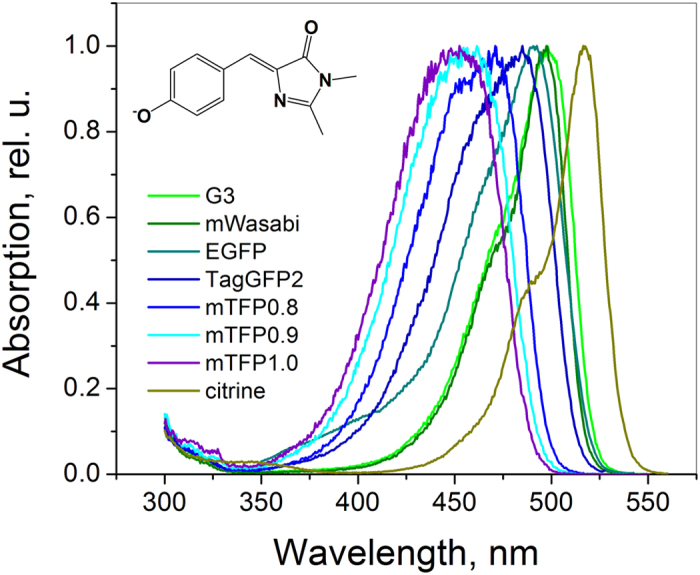
Normalized fluorescence excitation spectra of a series of FPs with the same, GFP-type chromophore in the anionic state.

**Figure 2:**
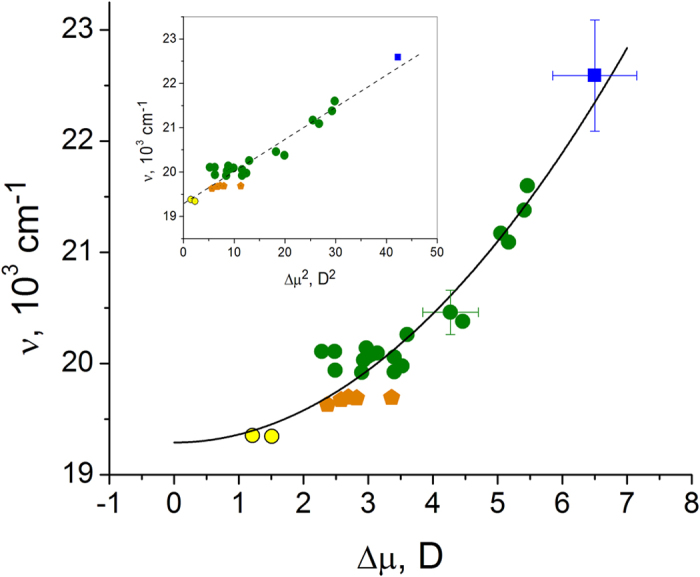
Dependence of the 0–0 transition frequency on the change of the permanent dipole moment for a GFP anionic chromophore in a number of different local environments. The blue square represents the model HBDI^−^ chromophore in alkaline D_2_O solution. Standard deviations are shown by bars. Twenty six different FP variants under investigation are sub-divided into three different groups, according to the local structure of their chromophore surrounding: (1) The mutants derived from EGFP and mTFP, where the two chromophore oxygen atoms, i.e. at phenolate and imidazolinone rings, maintain five hydrogen bonds with the surrounding, similar to the original EGFP and mTFP^63^,^64^,^65^,^66^, are shown by green circles; (2) EGFP-type of mutants containing, among others, the T203I mutation, which causes a reduction of the number of hydrogen bonds from five to four in the chromophore cluster, are shown by orange pentagons; (3) Yellow FP derivatives of GFP containing, among others, the T203Y mutation, which along with the reduction of the number of hydrogen bonds also causes a π-stacking interaction of the chromophore with the Tyr203 phenol^38^, are shown by yellow circles. The inset shows the same data in the linearizing, *ν* versus Δ*μ*^2^ coordinates.

**Figure 3:**
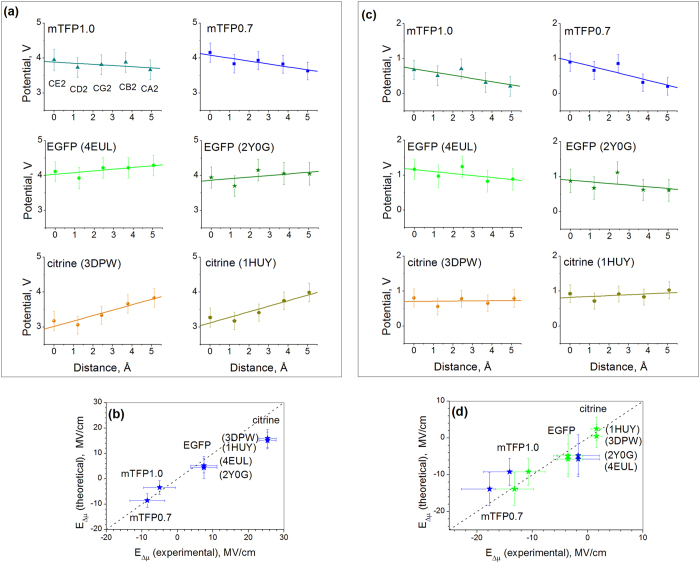
(**a**) MD-simulated electrostatic potentials on the chromophore atoms as a function of the distance along the straight line connecting CE2 and C2 atoms (see Fig. 4 for atom notations) for six selected FP structures. For EGFP and citrine, the two sets of data points correspond to two different pdb files. The linear regressions based on the potentials of the five atoms are shown by straight lines. (**b**) Comparison of experimentally measured electric fields with the fields obtained from the MD simulations of potentials shown in (**a**). Dashed line indicates exact coincidence. (**c**) Same as in (**a**) but when calculating potentials, in addition to the chromophore the contribution from five or four (in case of citrine) amino acids or water molecules in close proximity to the chromophore were also excluded. (**d**) Comparison of electric fields obtained from experimentally measured Δ*μ* values using the parameters Δ*μ*_HB_ and Δ*α* either obtained from experiment (blue symbols) or calculated (green symbols) with the fields obtained from the MD simulations of potentials shown in (**c**). Standard deviations are shown by bars in each panel.

Simple inspection of Fig. 1 suggests that the blue shift of absorption in the series of mutants is accompanied by systematic broadening of the spectrum. Since the more blue-shifted spectra correspond to larger Δ*μ* (Fig. 2), we hypothesized that the broadening is due to Stark-induced variations of transition frequencies in response to stochastic fluctuations of local fields. If we assume that the field’s fluctuations obey the normal distribution with the standard deviation δ*E*_Δ*μ*_ and that δ*E*_Δ*μ*_ does not vary much from one protein to another (which is supported by MD simulations, see Supplementary Tables S4 and S5), then larger Δ*μ* should produce a larger spread of transition frequencies. In this case, the field-dependent part of the spectral broadening, 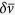, can be obtained by using Eqs. (3) and (2),


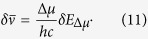


If, in addition to 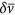, there is also a field-independent broadening, due to e.g. low-frequency vibrations, which is described by a Gaussian envelope with standard deviation *γ*, then the total linewidth of the 0–0 transition will read:


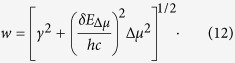


Figure 5 shows an experimental correlation of *w*^2^ and Δ*μ*^2^. From the slope and intercept of the linear regression, we obtain δ*E*_Δ*μ*_ = (5 ± 0.3) MV/cm and *γ* = 270 ± 25 cm^−1^, respectively. The first value compares well with the standard deviation of the fields fluctuations found in MD simulations (~6–7 MV/cm, see Supplementary Tables S4 and S5). The second value is close to the linewidth of the 0–0 transition of the bare chromophore in vacuum (310 ± 45 cm^−1^)^29^. This justifies our assumption that the protein-to-protein variations of spectral widths can be explained by the electric field-induced variations of the Stark shifts of individual transitions.

### Specific case: HBDI^−^ chromophore in water. Solvent reaction field E = E_R_

The 0–0 transition of the HBDI^−^ in D_2_O solution is significantly shifted to higher frequencies compared to all the proteins and the corresponding Δ*μ* value reaches the largest value, 6.5 D, Fig. 2. Several high-level quantum calculations predict similar Δ*μ* values for HBDI^−^ in water (Supplementary Table S1). The CASPT2/CASSCF method treating solvent molecules explicitly^16^,^22^ provides a very good agreement with the experiment. Simulation of the solvent with a polarized continuum model gives a slightly overestimated result, i.e. 8.6 D^24^. Our own quantum mechanical calculations show that the addition of five hydrogen-bonded water molecules to a bare chromophore (three at the phenolate oxygen and two at the imidazolinone oxygen, cf. Ref. 22) slightly increases Δ*μ*, from 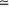
4.4 D (in vacuum, both experimental and theoretical) to 5.2 D. Therefore the increase of Δ*μ* from 4.4 D in vacuum (from 5.2 D in cluster) to Δ*μ* = 6.5 D in bulk water may be attributed to the effect of a polarizable water solution around the chromophore (or cluster). The corresponding part of the dipole moment change, Δ*μ*_*ind*_ = 2.1 D (1.3 D), induced by the reaction field *E*_*R*_ of the polarized solvent, can be presented as follows, cf. (1),


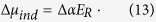


Here both Δ*μ*_*ind*_ and *E*_*R*_ represent the projections of the corresponding vectors on the direction of Δ*μ*_0_ (Δ*μ*_HB_). Using experimental values of Δ*α* = −35 Å^3^ and Δ*μ*_*ind*_ = 2.1 D (1.3 D), we find *E*_*R*_ = −18 MV/cm (−11 MV/cm).

For comparison, we also use the classical polarizable dielectric model to estimate the reaction field of the solvent. In this model, the field *E*_*R*_ is created by a chromophore with the ground-state dipole moment *μ*_g_ and polarizability *α*_g_, that is placed in the center of a spherical cavity with radius *a* possessing the internal dielectric constant *ε*_in_, which in turn is immersed in a solvent with the dielectric constant *ε*. The field is assumed to be the same for the ground and excited states of a molecule and is presented as^47^:


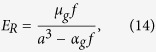


where *f* is the cavity field factor, described as^47^,^48^:


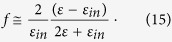


An estimation, using *ε* = 79 and *ε*_*in *_= 2^47^,^48^, as well as experimentally determined parameters (see Supplementary Discussion): *a *= 4.7 Å (for the cluster of the HBDI^−^ chromophore with 6 hydrogen-bonded water molecules), *α*_g _= 22 Å^3^, and literature value *μ*_g _= −11 ± 2 D^22^,^31^,^49^, yields *E*_*R *_= −17 MV/cm. The latter value agrees well with those obtained above from the field-induced dipole moment change in cluster (−11 MV/cm) and in vacuum (−18 MV/cm). Due to symmetry reasons, the field ***E***_*R*_ is directed along the *μ*_*g*_ vector. Since in HBDI^−^ chromophore the permanent dipole moment decreases upon excitation^20^,^50^, the Δ***μ*** is antiparallel to ***μ***_g_, and therefore ***E***_*R*_ is antiparallel to Δ***μ***. This means that ***E***_*R*_ found for water has the same direction (in the chromophore frame) and similar amplitude as the long-range field ***E***’_Δ*μ*_ inside the mTFP0.7 protein.

In conclusion, we have evaluated local electric fields in a series of fluorescent proteins with the same anionic GFP chromophore by using simultaneous measurements of 1PA and 2PA cross sections and the 0–0 transition frequency. In the first approximation, we considered the chromophore interacting with the rest of the protein and obtained the total effective protein field. In the second, - we considered a subset of FPs with similar local surrounding and selected a hydrogen-bonded cluster interacting with further-separated layers of the protein, which made it possible to obtain the long-range part of the field. Independent evaluations of the total and the long-range fields complement each other and provide the local, short-range field. We have shown that both total and long-range fields are well reproduced by MD simulations, which justifies our method of measurement. Our model quantitatively explains the spectral shifts of yellow and teal FPs versus EGFP as well as the spectral position of the isolated chromophore in water solution.

## Methods

### Measurements of Δ*μ*

The method of the measurement of Δ*μ* using two-photon absorption (2PA) spectroscopy was described previously^14^,^51^. Briefly, first the one-photon absorption spectrum (obtained in the forms of fluorescence excitation spectrum) of a protein was fitted with four or five Gaussians of the same width (three Gaussian fit was used for the HBDI- chromophore in D_2_O), see Supplementary Fig. S4. The width was systematically varied to obtain the minimum χ^2^. This resulted in the best fit of the 1PA spectrum, yielding three important parameters: spectral position of the lowest-energy transition *ν*_0–0_, its corresponding peak extinction coefficient *ε*(0–0), and its spectral width (Gaussian standard deviation) *w*. As a next step, the two-photon absorption spectrum presented as the σ_2_ value versus transition frequency (i.e. twice the laser frequency) was fitted to the same number of Gaussian peaks where the positions and amplitudes of the peaks were free, but the widths were fixed and equal to *w* (i.e. found from fitting of the 1PA spectrum). The two-photon cross section σ_2_(0–0), corresponding to the lowest-energy Gaussian peak was obtained from the best fit. The Δ*μ* value was then calculated as follows^14^:


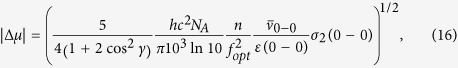


where *γ* is the angle between ***μ*** and Δ***μ*** vectors, which in the case of GFP chromophore is ≈ 20^0^ (see Ref. 52), *f*_*opt *_= (*n*^2 ^+ 2)/3 = 1.26 is the Lorentz local field factor calculated for water.

The two-level approximation used in the derivation of eq. (16) was justified by quantum chemical calculations of the sum-over-states expression of the 2PA cross section for HBDI- chromophore. For 30 different arrangements (including HBDI^−^ chromophore in vacuum and in different clusters representing local HB-surrounding of EGFP, TFP, and citrine mutants), ZINDO calculations of Δμ and 2PA cross sections show that the contribution of intermediate states higher than S_1_ to the σ_2_ value was not larger than 5% (average ~ 4%). A few contributions of 10–20% were found only when the 2PA was weak due to a small dipole change (Δμ ≤ 1 D).

The 1PA and 2PA spectra of all of the FPs studied here were published elsewhere^53^,^54^. The 2PA spectrum of the HBDI^−^ chromophore (Supplementary Fig. S4) was measured using the femtosecond nonlinear transmission (NLT) setup described in^55^. Fluorescein in water (pH11) was used for the 2PA standard (21.3 mM in 0.5 cm cuvette). The sample solution was a 10 cm path length of 19.6 mM HBDI^−^ in D_2_O + 0.1 M NaOD. The Rayleigh length of the beam for the measurement was > 50 cm for all wavelengths. The maximum two-photon cross section obtained for this sample (40 GM) is close to what was observed previously for a similar molecule^56^.

### Quantum calculations

**Figure 4:**
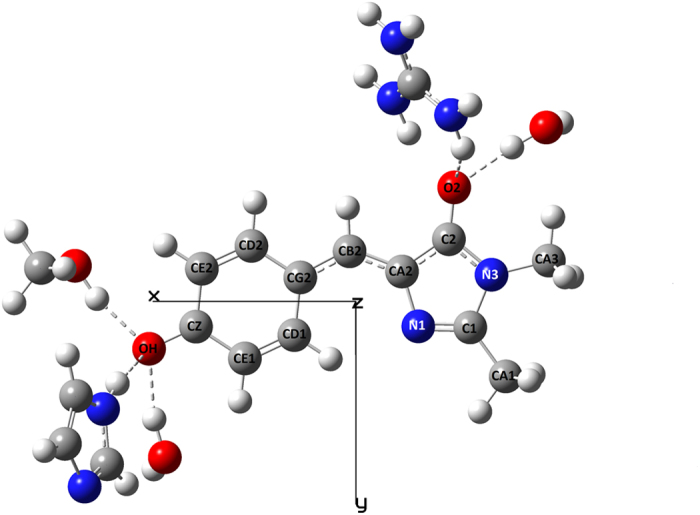
Schematic of the cluster, using mTFP0.7 structure (pdb file 2OTB) as an example that contains the HBDI^−^ chromophore and five hydrogen-bonded groups.

**Figure 5:**
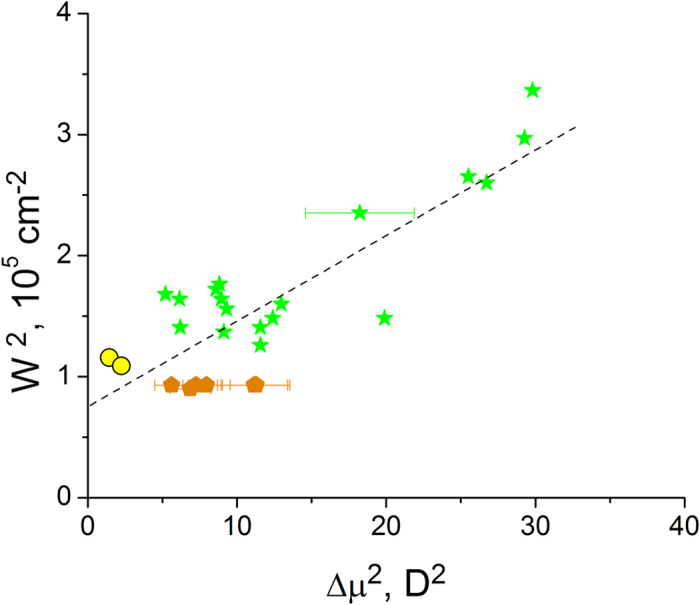
Dependence of the square of the spectral width (standard deviation of the Gaussian envelope, see Methods) of the 0–0 1PA transition on the square of the change of permanent dipole moment for a series of 26 GFPs. The colors of symbols are the same as in Fig. 2.

Beginning with the X-ray structure 2OTB.pdb for the mTFP 0.7 conformation, the chromophore and the surrounding residues His163, Ser 146, Wat 368, Arg 95, and Wat 361 were read into GaussView 5.08, with no alteration of coordinates except addition of H atoms. The resulting structures were truncated and terminated with methyl groups, yielding the structure in Fig. 4. The same approach was used for EGFP (4EUL.pdb) and citrine (3DPW.pdb) clusters. The geometry of the chromophore and any water molecules was optimized using m062x/6–31 g(d), while the non-water members of the cluster were frozen at the original pdb coordinates. CHARMM27^57^ point charges were placed at the resulting cluster atom coordinates, and Δ*μ* values, along with transition energies and transition moments, were obtained for the lowest 30 excited states from Zerner’s spectroscopically calibrated INDO/S2-CIS (ZINDO)^58^,^59^ with oxygen parameters from the Truhlar group^60^. 196 excited singlet configurations were included using Mataga-Nishimoto electron repulsion screening and the original CNDO/S overlap factors. The cluster was represented with point charges because we found that including the entire cluster in the ZINDO calculations introduced small amounts of contamination from low-lying charge transfer states involving the nearby positively charged Arg into the lowest excited state, S_1_. The large Δ*μ* values of the CT states made the computed S_0_ → S_1_ Δ*μ* values erratic and unreliable.

Optimizing the chromophore geometry in vacuum or in the cluster showed the resulting Δ*μ* to be reasonably insensitive to method and basis set, see Supplementary Tables S6 and S7.

To obtain the Δ*α* value, the dependence of Δ*μ* on *E*_Δ*μ*_ was calculated by applying a uniform electric field to the chromophore (Supplementary Fig. S1), using a combination of DFT and ZINDO. The geometry of the chromophore was optimized in the ground state using m062x/6–31 g(d) at each value of the field. The DFT+field-optimized geometries were then used in ZINDO calculations of Δ*μ* vs. field between −15 and 0 MV/cm, i.e. corresponding to the range of the actual fields. The slope of the resulting linear regression describing the Δ*μ* (*E*_Δ*μ*_) yields Δ*α* = −49 Å^3^, i.e. similar to experiment (−35 Å^3^). Note that the absolute Δ*α* values obtained from DFT methods using different functionals are 1.6–4 times smaller than the experimental one (Supplementary Table S2).

Starting from the corresponding PDB structure (citrine: 1HUY^61^, 3DPW^62^; EGFP: 2Y0G^63^, 4EUL^64^; mTFP0.7: 2OTB^65^; mTFP1: 2HQK^66^), the first chain was selected in case of dimer or tetramer complexes, preserving chrystallographic water molecules within 4 Å of the protein chain and hydrogen atoms were added by the pdb2gmx utility of Gromacs. Regarding the protonation state of some relevant residues, in citrine and EGFP the protonation state of Glu222 was set neutral, according to the accepted model, whereas all other Glu were considered anionic. All Histidine residues were set neutral, except His197 of mTFP0.7 and mTFP1, which was set cationic (protonated). In EGFP and citrine His25, 148, 169, 181, 199, and 217 were set as δ (i.e. with the proton in their δ nitrogen atom), and His77, 81, and 139 as ε (i.e. with the proton in their ε nitrogen atom). In mTFP0.7 and mTFP1 His25, 123, 163 and 204 (172, 173, 193) were set as δ (ε). The proteins were solvated in a truncated octahedron box of initial side length 75 Å, the complete systems containing, beside the protein, ~9500–9600 total water molecules, 20 Cl- ions and the following number of Na+ ions: 27 in 4EUL, 2Y0G and 1HUY, 23 in 2OTB, 22 in 2HQK, to neutralize the system. The different numbers of Na+ in the two citrine models 1HUY and 3DPW are due to the presence of the solvent exposed Q80R mutation in the former structure.

The amber99SB*-ILDN force field was used for the simulations^67^,^68^. The force field of the anionic chromophore was adapted from Ref. 69. Periodic boundary conditions were applied and electrostatics was treated using the smooth particle mesh Ewald method with a grid spacing of 1.2 Å and a 10 Å real-space cutoff. The simulation protocol consisted in initial 400 steps conjugate-gradient minimization with restraints on the protein non-hydrogen atoms (spring force 5 kJ mol^−1^ Å^−2^), followed by three 200-ps runs during which the restraints spring force constant was set at 5, 3 and 1 kJ mol^−1^ Å^−2^. In the following 2-ns production runs maintaining 1 kJ mol^−1^ Å^−2^ restraints, the system configurations were saved each ps. In all MD runs a 2 fs time step was used, LINCS was applied to constrains covalent bond lengths involving hydrogen atoms^70^, and the neighbor list is updated every 10 steps. Constant 300 K temperature and 1 kbar pressure were maintained by v-rescale thermostat^71^ (with a coupling of τ_T _= 1 ps) and Parrinello-Rahman barostat (τ_P _= 4 ps). The simulations were performed with the Gromacs 4.6.3 package^72^.

The electrostatic potential V at site **R** was calculated for each snapshot (disregarding the first 500 ps of simulation) using a homemade script as the sum of the contribution of each atom’s Amber partial charge *q*_*i*_, i.e.


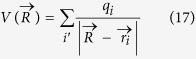


*r*_i_ being the position of atom *i*. The sum did not include either (a) the bare chromophore (atoms as in HBDI^−^ model, including up to the carbon atoms CA1 and CA3 of the methyl groups connected to the imidazolidinone ring), or (b) a cluster containing HBDI^−^ plus in citrine Gln94, Arg96, His148 and phenolate H-bonded water molecule; in EGFP same residues as in citrine plus Thr203; in the mTFPs Arg95,Ser146,His163, two water molecules one H-bonded to the phenolate, the other to the carbonyl of the imidazolidinone. Before calculating V the protein was centered in the box, and periodic boundary conditions were not accounted for, with the assumption that spurious surface effects are averaged out by the free-moving water molecules and ions. Comparison with the full Ewald sum calculation (using the PMEPot routine of VMD with an Ewald factor of 1 Å and a 0.2 Å grid^73^) was performed on one snapshot yielding relative errors smaller than 5%. The potentials at the chromophore atom positions as generated by all protein and water molecules (crystallographic and added to the simulation box) were averaged over the full simulation time (1.5 ns). Fluctuations (calculated as the standard deviation) of the electrostatic potential during MD were around 0.25 V, with a maximum of 0.38 V (on atom O2 of EGFP 4EUL).

## Additional Information

**How to cite this article**: Drobizhev, M. *et al.* Long- and Short-Range Electrostatic Fields in GFP Mutants: Implications for Spectral Tuning. *Sci. Rep.* **5**, 13223; doi: 10.1038/srep13223 (2015).

## Supplementary Material

Supplementary Information

## Figures and Tables

**Table 1:** Calculated components and magnitude of Δ ***μ*** in vacuum and different HB-clusters of the HBDI^−^ chromophore.

**No.**	**Chromophore**	**Environment**	**Δ*μ*_x_D**	**Δ*μ*_y_ D**	**Δ*μ*_z_ D**	**|Δ*****μ*****| D**
1	HBDI^−^	vacuum	4.30	0.66	0.11	**4.35**
2	HBDI^−^5 HBs (H_2_O)	P: 3 x H_2_O I: 2 x H_2_O	4.92	1.68	−0.16	**5.20**
3	HBDI^−^ 5 HBs (mTFP)	P: Ser146, His163, H_2_O I: Arg95, H_2_O	3.04	1.30	−0.01	**3.30**
4	HBDI^−^	vacuum, but chromophore geometry as in 3.	2.86	0.68	−0.01	**2.94**
5	HBDI^−^ 5 HBs (EGFP)	P: Thr203, His148, H_2_O I: Arg96, Gln94	2.82	1.10	0.02	**3.02**
6	HBDI^−^ 3 HBs, 4 groups (citrine)	P: His148, H_2_O I: Arg96, Gln94	1.55	0.80	−0.25	**1.76**

P: designates the phenolate oxygen local environment; I: designates the imidazolinone oxygen local environment. The entries represent: No. 1 - chromophore in vacuum; No. 2 - cluster (1), as in water solution; No. 3 - cluster (2) as in mTFP; No. 4 - chromophore in vacuum, but with the geometry optimized in cluster (2); No. 5 - cluster (3) as in EGFP; No. 6 - cluster (4) as in citrine. See text for description of cluster structures.

**Table 2:** Experimental and calculated model parameters, describing the transition frequency as a function of Δ*μ* in a series of FPs where the effective chromophore is presented as a cluster containing the HBDI^−^ molecule and five hydrogen-bonded groups, i.e. representing mTFP- and EGFP-derived mutants (see Fig. 4).

**Parameter**	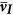 **cm**^−**1**^	***η***	***μ*, D**	**δ, Å^−3^**	**Δ*μ*_c_, D**	**Δ*μ*_*HB*_, D**
Experiment	20,620	0.57	6.9	0.033	3.69 ± 0.14	3.4 ± 0.2
Calculation (Ref.)	20,462 (20)	0.49 (20)	10.6 (this work)			3.0–3.3 (this work)

Definition of parameters is presented in the text. Standard deviation of Δ***μ***_c_ and Δ*μ*_*HB*_ are shown in the last two columns.
