# Supplementary material for: Long- and Short-Range Electrostatic Fields in GFP Mutants: Implications for Spectral Tuning
Source: Sci Rep. 2015 Aug 19;5:13223. doi: 10.1038/srep13223 (PMC4541067; doi:10.1038/srep13223)
Supplement: Supplementary Information [file srep13223-s1.pdf]

Supplementary Information

Long- and Short-Range Electrostatic Fields in GFP Mutants: Implications for Spectral Tuning

M. Drobizhev, P. R. Callis, R. Nifosì, G. Wicks, C. Stoltzfus, L. Barnett, T. E. Hughes, P. Sullivan, A. Rebane

Supplementary Table S1. The literature results of quantum calculations of $\Delta\mu$ for the p-hydroxybenzylidene-2,3-dimethyl-imidazolinone (HBDI$^-$) and its analogs: p-hydroxybenzylidene-imidazolinone (HBI$^-$), p-hydroxybenzylidene-2-methyl-imidazolinone (HBMI$^-$), and p-hydroxybenzylidene-2-methyl-imidazolinone-3-acetate (HBMIA$^-$) in vacuum, in water, and in GFP surrounding.

Chromophore	Environment	$\lvert\Delta\mu\rvert$	$\Delta\mu_x$	$\Delta\mu_y$	Method	Reference
VACUUM						
HBI$^-$	vacuum	1.0	+		CASPT2//CASSCF	1
HBI$^-$	vacuum	0.9	+		CASSCF-(16,14)*	2
HBI$^-$	vacuum	1.7			MS-MRPT2	3
HBI$^-$	vacuum	4.2	+		CASSCF-(12,11)	2
HBDI$^-$	vacuum	4.31	4.30	0.22	ZINDO	This work
HBDI$^-$	vacuum	2.0	+		CASPT2	4
HBDI$^-$	vacuum	1.7	+		CASSCF-(16,14)*	2
HBDI$^-$	vacuum	1.6	+		CC2	5
HBDI$^-$	vacuum	2.3	+		SORCI	5
HBDI$^-$	vacuum	1.5	+		OM2/MRCI	5
HBDI$^-$	vacuum	1.2			LC-BLYP	2
HBDI$^-$	vacuum	4.4	+		CASSCF-(12,11)	2
HBDI$^-$	vacuum	2.8			DFT/BLYP	2
HBDI$^-$	vacuum	1.7			B3LYP	2
HBDI$^-$	vacuum	1.2			CAM-B3LYP	2
HBDI$^-$	vacuum	2.7	+		TD-PBE	5
HBDI$^-$	vacuum	1.7	+		TD-B3LYP	5
HBMI$^-$	vacuum	2.15	2.11	0.43	SAC-CI//B3LYP	6
HBDI$^-$	vacuum	2.0	+		CASSCF-(12/11)/6-31G*	7
HBMIA$^-$	vacuum	2.93	+		CASPT2	4
HBMIA$^-$	vacuum	1.93	+		TD-DFT	8
HBDI$^-$	vacuum	2.34	+		ZINDO	9
HBDI$^-$	vacuum	2.87	+		BLYP	10
HBI$^-$	vacuum	1.5			CASSCF-(12,11)	2
HBDI$^-$	vacuum	1.4			CASSCF-(14,13)	2
HBDI$^-$	vacuum	0.7			MP2/cc-pVDZ (ground) CIS/6-31G* (excited)	11
HBI$^-$	vacuum	3.5			OM2/PERTCI	12
WATER						
HBDI$^-$	liquid D_2O + NaOD	6.5			experiment	This work

HBDI$^-$	water	8.6	+		CASSCF-(12/11)/6-31G*+PCM (implicit, PCM)	7
HBI$^-$	water	6.6	+		CASPT2/CASSCF/CHARMM (explicit 701 waters + Na$^+$)	13
HBDI$^-$	water	6.3			CASPT2 (Monte Carlo explicit 857 waters)	4
PROTEIN						
EGFP-chromophore	EGFP	3.5			experiment	This work
wt-GFP chromophore	GFP B form	6.8/f<br>f = 1.1-1.8<br>$\Delta\mu$=3.8-6.2			experiment	14<br>15
same	GFP S65T	7.0/f<br>f = 1.1-1.8<br>$\Delta\mu$=3.9-6.4			experiment	14<br>15
same	GFP_I_form	4.3	+		CASPT2/CASSCF/CHARMM	13
same	GFP_B_form	5.4	+		CASPT2/CASSCF/CHARMM	13
same	(1EMG (S65T) with T65S back and protonated Glu222)	5.0			CASSCF-(12,11)	2
same	(1EMG (S65T) with T65S back and protonated Glu222)	5.8	+		CASSCF-(16,14)	2
same	same	2.6			DFT/BLYP	2
same	same	1.5			B3LYP	2
same	same	2.0			CAM-B3LYP	2
same	same	2.8			LC-BLYP	2
same	1W7S with the proton transferered from Tyr66 to Glu222 and reorienting Thr203 to H-bond with chromophore	0.6	-		TD-PBE	5
same	same	0.5	+		TD-B3LYP	5
same	same	3.8	+		CC2	5
same	same	4.6	+		SORCI	5
same	same	5.1	+		OM2/MRCI	5

Sign + in the $\Delta\mu_x$ column designates the calculated positive direction of $\Delta\boldsymbol{\mu}$, i.e. from imidazolinone center to phenol center, sign – designates an opposite direction.

Supplementary Table S2. Experimental and theoretical values of the change of polarizability $\Delta\alpha$ (main component of the tensor) of the model GFP chromophores (abbreviations of chemical structures are the same as in Table1).

Chromophore	Environment	$\Delta\alpha_{xx}$ Å^3	geometry	Method	Reference
GFP series	extrapolated to vacuum	-35	in proteins	Experiment	This work
HBDI$^-$	vacuum	-13	Optimized with DFT-B3LYP/6-31G	ZINDO (for μ_1) Gaussian03	9
HBMIA$^-$	vacuum	-22	6-31G+(d,p)	DFT and CIS Gaussian03	8
HBDI$^-$	vacuum	-7.8	optimized without field	DFT cam-B31LYP/6-31G*	This work
HBDI-	vacuum	-12	optimized without field	DFT m11/6-31G*	This work
HBDI-	vacuum	-9.0	optimized without field	DFT M062X/6-31G*	This work
HBDI-	vacuum	-17	optimized for each field	DFT M062X/6-31G*	This work
HBDI-	vacuum	-49	optimized for each field and calculated at $\Delta\mu$ = 2.7 D	ZINDO	This work
HBDI$^-$	2OTB HB-cluster	-12.3	optimized without field	DFT M062X/6-31G* (?)	This work
HBDI$^-$	vacuum	-8.5	optimized in 2OTB HB-cluster	DFT M062X/6-31G* (?)	This work

Supplementary Table S3. Experimental parameters for the investigated set of GFP mutants with anionic chromophore and the model chromophore HBDI$^-$ in alkaline D_2O.

Protein	$\varepsilon_{0\text{-}0}$ $M^{-1}cm^{-1}$	$\nu_{0\text{-}0}$ cm^{-1}	σ_2(0-0) GM	$\Delta\mu$ D	w, cm^{-1}	$E_{\Delta\mu}$ MV/cm	$E'_{\Delta\mu}$ (exp) MV/cm	$E'_{\Delta\mu}$ (mod) MV/cm
mTFP0.7	36500	21600	31	5.45	580	-8.4	-17.6	-13.2
mTFP0.8	41900	21090	33	5.2	510	-6.3	-15.4	-11.6
mTFP0.9	40400	21380	31	5.4	545	-8.0	-17.1	-12.9
mTFP1.0	44100	21170	33	5.05	515	-5.0	-14.1	-10.7
G1	67600	20380	41	4.5	385	-0.26	-9.4	-7.3
G3	59800	19920	26	3.4	375	9.2	0	-0.61
mWasabi	67050	20060	24	3.4	355	9.2	0	-0.61
EGFP	40300	20260	15		500	7.5	-1.7	-3.6
TagGFP2	29900	20460	16	4.3	485	1.5	-7.7	-7.8
citrine	69000	19340	5.0	1.5	330	25.4	-	1.59
EYFP	48800	19350	2.3	1.21	340	27.9	-	3.37
EGFP D117G,V163A	42070	20110	6.7	2.28	410	18.8	9.6	4.5
EGFP T203I	52250	19680	9.2	2.37	305	18	-	-3.7
EGFP V163A, Q184R	42350	20110	8.0	2.48	405	17.1	7.9	3.3
EGFP S72G, D117G,V163A	49500	19940	9.5	2.49	375	17	7.8	3.2
EGFP V163A, T203I	52250	19670	11.2	2.62	300	15.9	-	-5.3
EGFP S72G,T203I	52300	19700	11.8	2.69	305	15.3	-	-5.7
EGFP V68M,V163A,T203I	52250	19690	13	2.82	305	14.1	-	-6.5
EGFP N121S,V163A	39050	20100	10.3	2.93	415	13.2	4.0	0.55
EGFP V68M	41800	20140	11.3	2.97	420	12.9	3.7	0.31
EGFP D117G,V163A,S202N ,V219I	42900	20060	11.8	2.99	405	12.7	3.5	0.18
EGFP S72G,V163A	48950	19920	13.8	3.02	370	12.4	3.3	0
EGFP V163A	41250	20080	11.8	3.05	395	12.2	3	-0.18
EGFP Q80R,D117G,V163A, T203I	52250	19690	18.2	3.34	305	9.7	-	-9.7
EGFP D117G,V163A,T203I	52250	19690	18.4	3.36	305	9.5	-	-9.8
EGFP S72G	46200	19980	17.6	3.52	385	8.1	-1.03	-3.1
HBDI$^-$ in D_2O	22420	22590	26	6.5	1050	-17		

Parameters of the lowest-frequency vibronic transition: extinction coefficient, $\varepsilon_{0\text{-}0}$, maximum wavenumber, $\nu_{0\text{-}0}$, 2PA cross section, σ_2(0-0), and Gaussian standard deviation, w. $\Delta\mu$ is the dipole moment difference between the states S_1 and S_0. $E_{\Delta\mu}$ is the total effective field, $E'_{\Delta\mu}$ (exp) is the long-range field evaluated using all experimental paramerters; $E'_{\Delta\mu}$ (mod) is the long-range field evaluated using theoretically calculated $\Delta\mu_{HB}$ and $\Delta\alpha$.

Supplementary Table S4. Potentials on the chromophore atoms, calculated as the average during various MD trajectories. Electrostatic potential is given in V, standard deviations in parentheses. The chromophore charges were discarded.

	mTFP0.7 2OTB	mTFP1 2HQK	EGFP 4EUL	EGFP 2Y0G	Citrine 3DPW	Citrine 1HUY
OH	6.00(0.36)	5.60(0.42)	5.90(0.34)	5.86(0.39)	4.76(0.40)	4.78(0.37)
CZ	4.86(0.28)	4.43(0.32)	4.69(0.27)	4.72(0.32)	3.74(0.31)	3.81(0.30)
CE1	4.26(0.26)	3.77(0.32)	4.06(0.28)	4.30(0.35)	3.40(0.30)	3.43(0.30)
CD1	3.85(0.27)	3.58(0.30)	4.09(0.29)	4.33(0.32)	3.33(0.27)	3.36(0.27)
CG2	3.94(0.26)	3.82(0.29)	4.23(0.29)	4.17(0.31)	3.35(0.25)	3.42(0.25)
CD2	3.85(0.26)	3.74(0.29)	3.94(0.31)	3.71(0.30)	3.07(0.26)	3.19(0.25)
CE2	4.17(0.28)	3.96(0.30)	4.13(0.28)	3.96(0.30)	3.18(0.28)	3.28(0.27)
CB2	3.84(0.26)	3.89(0.28)	4.24(0.30)	4.07(0.32)	3.67(0.26)	3.76(0.25)
CA2	3.64(0.25)	3.68(0.28)	4.30(0.30)	4.06(0.32)	3.85(0.27)	4.00(0.27)
N2	2.95(0.26)	2.94(0.28)	4.25(0.35)	3.97(0.38)	3.85(0.30)	4.14(0.31)
C1	2.57(0.26)	2.61(0.27)	3.90(0.28)	3.77(0.32)	3.49(0.27)	3.84(0.28)
N3	3.69(0.26)	3.78(0.27)	4.34(0.27)	4.20(0.28)	4.19(0.27)	4.48(0.27)
C2	4.13(0.27)	4.23(0.28)	4.39(0.29)	4.25(0.29)	4.22(0.27)	4.43(0.27)
O2	5.36(0.33)	5.48(0.33)	5.17(0.38)	5.12(0.33)	5.14(0.33)	5.29(0.33)

Supplementary Table S5. Potentials on the chromophore atoms, calculated as the average during various MD trajectories. Electrostatic potential is given in V, standard deviations in parentheses. The chromophore and hydrogen bonded amino acids (5 for mTFPs and EGFPs and 4 for citrines) charges were discarded.

	mTFP0.7 2OTB	mTFP1 2HQK	EGFP 4EUL	EGFP 2Y0G	Citrine 3DPW	Citrine 1HUY
OH	1.28(0.27)	0.95(0.32)	1.43(0.29)	1.33(0.30)	1.41(0.30)	1.37(0.30)
CZ	1.38(0.26)	0.97(0.29)	1.40(0.27)	1.32(0.30)	1.25(0.27)	1.30(0.27)
CE1	1.56(0.26)	1.06(0.32)	1.33(0.29)	0.88(0.28)	1.23(0.28)	1.29(0.27)
CD1	1.19(0.26)	0.91(0.30)	1.43(0.30)	0.68(0.28)	1.11(0.26)	1.18(0.25)
CG2	0.86(0.25)	0.71(0.29)	1.25(0.31)	1.13(0.30)	0.79(0.24)	0.92(0.23)
CD2	0.66(0.26)	0.51(0.28)	0.97(0.33)	1.59(0.32)	0.56(0.24)	0.72(0.23)
CE2	0.90(0.26)	0.68(0.28)	1.17(0.29)	1.49(0.34)	0.81(0.26)	0.93(0.25)
CB2	0.32(0.25)	0.32(0.28)	0.83(0.31)	0.63(0.30)	0.65(0.24)	0.83(0.23)
CA2	0.21(0.25)	0.21(0.29)	0.89(0.31)	0.61(0.31)	0.79(0.25)	1.03(0.25)
N2	0.22(0.25)	0.21(0.29)	1.46(0.36)	1.17(0.38)	1.39(0.30)	1.74(0.30)
C1	-0.02(0.26)	0.01(0.27)	1.21(0.30)	1.05(0.32)	1.07(0.26)	1.42(0.27)
N3	0.57(0.26)	0.66(0.27)	1.12(0.29)	0.92(0.28)	1.23(0.25)	1.50(0.26)
C2	0.06(0.25)	0.15(0.26)	0.35(0.29)	0.13(0.27)	0.49(0.25)	0.78(0.24)
O2	-0.11(0.27)	-0.01(0.27)	-0.01(0.39)	-0.27(0.30)	0.15(0.26)	0.46(0.25)

Supplementary Table S6. Effect of method and basis set on optimization of HBI anion in vacuum.

Method/Basis	S_0-S_1 transition frequency cm^{-1}	S_0-S_1 transition wavelength nm	$\Delta\mu$	$\Delta\mu_x$	$\Delta\mu_y$	$\Delta\mu_z$	Oscillator strength
m06L/6-311+G(2df)	22660	441.30	4.51	4.48	0.51	0.084	1.20
b3lyp/6-311+G(2df)	22672	441.06	4.54	4.51	0.52	0.085	1.20
HF/3-21g	23071	433.45	4.33	4.33	0.03	-0.02	1.19
b3lyp/6-31g(d)	22494	444.55	4.30	4.30	0.12	-0.03	1.21
m062x/6-31g(d)	22647	441.57	4.36	4.30	0.66	0.11	1.20
m06L/6-31g(d), old O	21876	457.12	4.25	4.16	0.88	-0.06	1.31
m06L/6-31g(d)	22526	443.93	4.37	4.37	0.12	-0.03	1.21
FilippiDFT-BLYP-methyls[a]	22342	447.58	4.42	4.38	-0.60	0.00	1.21
FilippiDFT-BLYP-H [a]	22384	446.74	4.31	4.30	-0.29	0.00	1.20

[a] Ref. 2.

Supplementary Table S7. Comparison of Some Cluster Geometries and Optimization Methods

Item	Chromophore	Environment	$\Delta\mu_x$, D	$\Delta\mu_y$, D	$\Delta\mu_z$, D	$\lvert\Delta\mu\rvert$, D
1	2OTB original pdb coordinates[a]<br>5 HBs: hf/3-21g	P: Ser, His, H_2O,<br>I: Arg, H_2O	2.95	0.78	0.06	**3.05**
2	5 HBs: hf/3-21g [b]	P: Ser, His, H_2O,<br>I: Arg, H_2O	2.9	0.86	0.02	**3.02**
3	5 HBs: m062x/6-31g(d)[c]	P: Ser, His, H_2O,<br>I: Arg, H_2O	3.04	1.30	-0.01	**3.30**
4	hf/3-21g	vacuum, but chromophore geometry as in 3	2.60	0.23	0.00	**2.61**
5	m062x/6-31g(d)	vacuum, but chromophore geometry as in 4	2.95	0.19	0.24	**2.97**
6	5 HBs: hf/3-21g[d]	P: Thr, His, H_2O,<br>I: Arg, Gln	3.01	0.83	0.02	**3.12**
7	5 HBs: m062x/6-31g(d)[e]	P: Thr, His, H_2O,<br>I: Arg, Gln	2.82	1.10	0.02	**3.02**
8	4 HBs: hf/3-21g[f]	P: His, H_2O<br>I: Arg, H_2O	0.94	0.66	0.04	**1.15**
9	4 HBs: m062x/6-31g(d)[g]	P: His, H_2O<br>I: Arg, H_2O	1.64	0.72	0.03	**1.79**

[a]No optimizing. Chromophore bonds were adjusted to those of item 2

[b]Entire cluster + chromophore optimized; $\Delta\mu$ calculated with point charge environment

[c]Chromophore and waters optimized; non-water cluster frozen; $\Delta\mu$ calculated with point charge environment

[d]Environment point charges as same as item 3 but Q94 inserted in place of H_2O near Arg 96

[e]Chromophore and water optimized; non-water cluster frozen; $\Delta\mu$ calculated with point charge environment

[f]All optimized except His; $\Delta\mu$ calculated with point charge environment

[g]All non-chromophore atoms frozen; $\Delta\mu$ calculated with point charge environment

Supplementary Discussion.

1. Derivation of Eqs. (6)-(10).

For independent experimental evaluation of $\Delta\mu_{HB}$, and consequently $E'_{\Delta\mu}$, we consider here the effects of both short- and long-range interactions on the pure-electronic electronic transition frequency ($\nu_{0\text{-}0}$) of the chromophore. Our goal is to find a correlation between the two observables: $\nu_{0\text{-}0}$ and $\Delta\mu$. We proceed in two steps: (1) we first consider the effects of the chromophore structural changes (due to both short- and long-range electrostatic interactions), and (2) add the electronic contributions to the energy, described in terms of the Stark shifts (cf. approach in Ref. 16).

The main structural perturbation occurring upon "dressing" the initially bare chromophore with the electrostatic surrounding involves the change of alternating single and double bond lengths. (Other degrees of freedom, e.g. twisting with the respect to the bridge bonds, can be disregarded because the crystallography data demonstrate nearly planar geometry of the chromophore for a representative set of mutants, i.e. mTFP 0.7, mTFP 1.0, EGFP, and citrine.) To describe the effect of bond length alterantion quantitatively, we use the two-forms two-states (2F2S) model of resonating forms[16-18], which was recently adapted to the GFP-type of chromophores in Refs. 19-22. In this model, the molecular state of the chromophore can be described as a linear combination of the two limiting resonating forms (both with a definite, but reversed order of single and double bonds). Upon application of the field (either short- or long-range), the real structure can be continuously tuned from one form, with zero dipole moment, to another form, with maximum dipole moment. The resulting dipole moment difference, $\Delta\mu_{\mathrm{BLA}}$, is a linear function of the bond-length alternation parameter, which becomes 0 in the middle of the tuning range (so-called "cyanine limit"). Therefore, the change of the permanent dipole moment presents a good sensor not only for a long-range uniform field, but also for the structural changes which can be due to both short- and long-range interactions.

The 2F2S model predicts the following dependence of optical transition energy, $h\nu_{\mathrm{LR}}$, (subscripts L and R denote, respectively, left and right parts of the GFP chromophore, connected by a methine bridge) on the change of dipole moments ($\Delta\mu_{\mathrm{BLA}}$)[3]:

$$h\nu_{LR} = h\nu_I \left(1 + \frac{\Delta\mu_{BLA}^2}{\eta^2 M^2 (1 - \Delta\mu_{BLA}^2 / M^2)}\right)^{1/2}, \qquad (1)$$

where

$$h\nu_I = 2\left(\frac{1}{h\nu_{LL}} + \frac{1}{h\nu_{RR}}\right)^{-1} \qquad (2)$$

is the harmonic mean of the excitation energies of the symmetric parent molecules, i.e. those carrying the same nucleus on both sides (either two phenol rings or two imidazolinone rings in the case of GFP chromophore). η is the dimensionless parameter whose physical meaning consists in the "screening" of the charge-transfer transition via redistribution of those electrons clouds which are not directly involved in the transfer[3,23]. Its typical value varies from 0.5 to 1, Ref. 3. Note that in the simplified version of the

model, i.e. without screening, parameter η was considered to be equal to 1, Refs. 18,24. In eq. (1), M denotes the diabatic molecular dipole (i.e. obtained by very slow transfer of an electron from one side of the molecule to another):

$$M = \sqrt{\Delta\mu_{BLA}^2 + 4\mu^2} \ , \tag{3}$$

where μ is the transition dipole moment between the ground and excited states. Parameter $\Delta\mu_{BLA}$ is defined such that $\Delta\mu_{BLA} = x\,M$, where is x the bond length alternation (BLA). For simple one-dimensional polyene molecules, $\Delta\mu_{BLA}$ is equal to the observed $\Delta\mu$, and $\Delta\mu_{BLA} = \Delta\mu = 0$ when all C-C bonds of the chain are of the same length (cyanine limit). Because the GFP chromophore is not as simple as linear polyene, i.e. having heterogeneous atoms and aromatic rings in the conjugation path, the bond-length alternation considered at one site of the molecule, e.g. in the methine bridge, is not an ideal parameter[3,19,22]. This means that observed $\Delta\mu$ can be non-zero at $x = 0$. To account for this effect, we define $\Delta\mu_{BLA}$ as follows[20]:

$$\Delta\mu = \Delta\mu_c + \Delta\mu_{BLA} \ , \tag{4}$$

where $\Delta\mu_c$ is a constant, corresponding to $x = 0$. Generally, and in the case of GFP in particular, $\Delta\mu^2_{BLA} << M^2$, and therefore, eq. (1) can be simplified to give:

$$h\nu_{LR} \approx h\nu_I + \frac{\Delta\mu_{BLA}^2}{2\eta^2 M^2} h\nu_I \approx h\nu_I + \frac{\Delta\mu_{BLA}^2}{8\eta^2\mu^2} h\nu_I \, . \tag{5}$$

Substituting (4) into (5), we finally get the following expression for the dependence of transition energy on the structural changes, reflected by the permanent dipole difference:

$$h\nu_{LR} \approx h\nu_I + \frac{(\Delta\mu - \Delta\mu_c)^2}{8\eta^2\mu^2} h\nu_I \, . \tag{6}$$

Our next step is to include the electronic (Stark effect) interaction of the chromophore with the long-range quasi-uniform field $E'_{\Delta\mu}$, created predominantly by charged amino acid residues outside of the inner hydrogen-bonded cluster. Note that we disregard any short-range electronic interactions and possible changes of the field upon excitation of the chromophore, e.g. through polarizability of surrounding. Since $\Delta\mu$ depends on the field itself, the Stark effect corrections to the Hamiltonian should be considered to the second order[25]. Adding the corresponding terms (as a perturbation) to the energies of the ground and excited states, we obtain instead of (6):

$$h\nu_{LR} = h\nu_I + \frac{(\Delta\mu - \Delta\mu_c)^2}{8\eta^2\mu^2} h\nu_I - \Delta\mu_{HB} E_{\Delta\mu} - \frac{1}{2}\Delta\alpha E_{\Delta\mu}^2 \, . \tag{7}$$

To keep only the experimentally observable values, i.e. $\Delta\mu$ and $h\nu_{LR}$, we substitute $E_{\Delta\mu}$ for $\Delta\mu$, using (2) of the main text, and obtain:

$$h\nu_{LR} = h\nu_I + \frac{(\Delta\mu - \Delta\mu_c)^2}{8\eta^2\mu^2} h\nu_I - \frac{1}{2\Delta\alpha}(\Delta\mu^2 - \Delta\mu_{HB}^2), \tag{8}$$

which can be re-written in a standard form of the second-order polynomial as follows

$$h\nu_{LR} = \left(\delta - \frac{1}{2\Delta\alpha}\right)\Delta\mu^2 - 2\delta\Delta\mu_c\Delta\mu + \left(h\nu_I + \frac{\Delta\mu_{HB}^2}{2\Delta\alpha} + \delta\Delta\mu_c{}^2\right), \tag{9}$$

where we used a notation $\delta = \frac{h\nu_I}{8\eta^2\mu^2}$. If $\Delta\mu_{HB}$ = const, as it can be expected for a series of proteins with similar HB-environment, then (9) will present a second order polynomial for the transition frequency as a function of $\Delta\mu$. Transition to wavenumbers, $\nu = c\bar{\nu}$, where c is the speed of light, yields:

$$\bar{\nu} = \frac{1}{hc}\left(\delta - \frac{1}{2\Delta\alpha}\right)\Delta\mu^2 - \frac{2\delta\Delta\mu_c}{hc}\Delta\mu + \left(\bar{\nu}_I + \frac{\Delta\mu_{HB}^2}{2hc\Delta\alpha} + \frac{\delta\Delta\mu_c{}^2}{hc}\right) \tag{10}$$

Equation (10) is the main results of the model, which will be used to describe the experimental data and to obtain the long-range electric fields. Equation (10) predicts that the optical transition frequency is described by a second-order polynomial of the dipole moments difference, i.e.

$$\bar{\nu} = A\Delta\mu^2 + B\Delta\mu + C, \tag{11}$$

where

$$A = \frac{1}{hc}\left(\delta - \frac{1}{2\Delta\alpha}\right), \tag{12}$$

$$B = -\frac{2\delta\Delta\mu_c}{hc}, \tag{13}$$

$$C = \left(\bar{\nu}_I + \frac{\Delta\mu_{HB}^2}{2hc\Delta\alpha} + \frac{\delta\Delta\mu_c{}^2}{hc}\right). \tag{14}$$

2. Model parameters.

Taking $\Delta\alpha$ = - 35 Å^3 from the experiment (see above), we obtain δ = 3.31 x 10^{22} cm^{-3} from eq. (12). Now using the definition, $\delta = \frac{E_I}{8\eta^2\mu^2}$ and assuming $\bar{\nu}_I \approx$ 20,000 cm^{-1}, i.e. the value close to the average transition frequency (see below), and μ = 6.9 D (average number for a series of mutants, Table 2 of the main text) we estimate η = 0.57, which is close to the theoretical value of 0.49 calculated by Olsen and McKenzie for the GFP class of chromophores[3].

Solving eq. (13) with the known δ, we find $\Delta\mu_c = 4.2 \pm 0.9$ D. By definition, parameter $\Delta\mu_c$ corresponds to the dipole moment difference when the BLA parameter equals to 0. Figure 2 presents the dependence of the measured $\Delta\mu$ on the calculated effective BLA parameter, extracted from a principal component analysis of chromophore structures in a balanced set of HDBI$^-$-containing FPs, including both the red-shifted YFP and the blue-shifted mTFP0.7[22]. This BLA parameter is a linear combination of the different bond lengths with the optimum weights and was calculated[22] for QM/MM optimized chromophore structures embedded in the pdb models of YFP (close analogue to citrine), S65T (close analogue to EGFP) and mTFP0.7.

The linear correlation between $\Delta\mu$ and effective BLA provides the value of $\Delta\mu_c = 3.7 \pm 0.1$ D, which is in good agreement with the above pure experimental value and will be used in calculation of $\Delta\mu_{HB}$ below.

To obtain $\Delta\mu_{HB}$, we first use eq. (10) for the free chromophore in vacuum where it has the 0-0 transition frequency equal to $\bar{\nu}_0$. Substituting $\Delta\mu = \Delta\mu_0 = 4.5$ D, and $E'_{\Delta\mu} = 0$ into (10) we find

$$\frac{E_I}{hc} + \frac{\delta(\Delta\mu_0 - \Delta\mu_c)^2}{hc} = \bar{\nu}_0 . \qquad (15)$$

where $\bar{\nu}_0 = 20{,}725$ cm^{-1} was measured using ionization spectroscopy in gas phase[26].

Second, we use eq. (14) for the subset of mutants with 5 HBs,

$$\frac{E_I}{hc} + \frac{\Delta\mu_{HB}^2}{2hc\Delta\alpha} + \frac{\delta\Delta\mu_c{}^2}{hc} = C \quad , \qquad (16)$$

where parameter C is known experimentally. Subtracting (15) from (16) and solving for $\Delta\mu_{HB}$, we arrive at

$$\Delta\mu_{HB} = \left(2hc\Delta\alpha\,\Delta\bar{\nu} + 2\delta\Delta\alpha\,\Delta\mu_0\left(\Delta\mu_0 - 2\Delta\mu_c\right)\right)^{1/2} , \qquad (17)$$

where $\Delta\bar{\nu} = C - \bar{\nu}_0 = 1335$ cm^{-1}. Substitution of all known parameters, results in $\Delta\mu_{HB} = 3.4 \pm 0.2$ D.

3. *Evaluation of the cavity radius a and the ground state polarizability α_g for the HBDI$^-$ chromophore in alkaline water solution*

We assume a to be the radius of the sphere in water solution occupying the same volume as does the cluster of HBDI$^-$ with 6 hydrogen-bonded water molecules[4]. The crystallographic volume of mono-methylated HBI$^-$ chromophore, hydrated with 3 water molecules is $V = 310$ Å^3, Ref. 27. Adding the van der Waals volume of one methane molecule, simulating the second methyl group of HBDI$^-$, (37 Å^3 , Ref. 28) and that of 3 extra water molecules (3 x 30 = 90 Å^3), we obtain $V = 437$ Å^3, and consequently, $a = 4.7$ Å. To obtain α_g, we use the perturbation theory expression for polarizability of state n[25]:

$$\alpha_g = 2\sum_i \frac{|\mu_{gi}|^2}{E_i - E_g} , \qquad (18)$$

where μ_{gi} is the transition dipole moment between states g and i, and E_i and E_g are the energies of the states i and g, respectively. The absorption spectrum of $HBDI^-$ chromophore consists of a single strong electronic transition, $S_0 \rightarrow S_1$, which allows using two-level approximation, involving only the ground (g) and excited (e) states to obtain:

$$\alpha_g = 2\frac{|\mu_{ge}|^2}{h\nu_{ge}} , \tag{19}$$

where μ_{ge} is the $S_0 \rightarrow S_1$ transition dipole moment and $h\nu_{ge}$ is the corresponding transition energy. The $|\mu_{ge}|^2$ value was obtained by integrating the absorption spectrum, according to the Strickler-Berg relation as follows[29]:

$$|\mu_{ge}|^2 = \frac{3\times10^3 \ln 10\, n\, hc}{8\pi^3 f_L^2 N_A}\int\frac{\varepsilon(\lambda)d\lambda}{\lambda} , \tag{20}$$

where n is the refractive index of the medium, $f = \frac{n^2+2}{3}$ is the Lorentz local field factor, $\varepsilon(\lambda)$ is the spectral dependence of extinction coefficient on wavelength, N_A is the Avogadro number. Substituting n = 1.333 for water and integrating the absorption spectrum of $HBDI^-$ in alkaline solution, we obtain $|\mu_{ge}|$ = 7.25 D in water. Using this value with $\bar{\nu}_{ge}$ = 23,800 cm^{-1} gives α_g = 22 $Å^3$.

Supplementary Figure S1. Quantum chemical calculation of the dependence of the dipole moment difference $\Delta\mu$ on the applied uniform field.

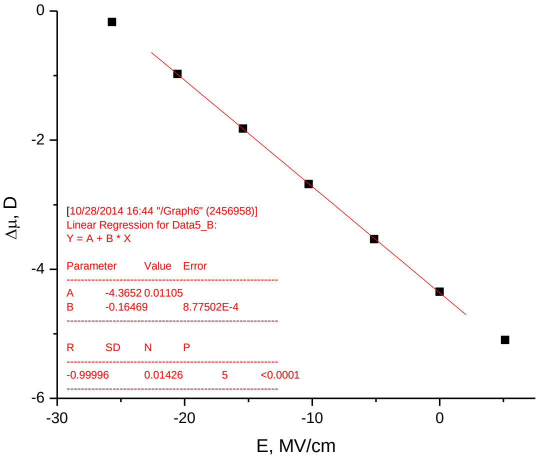

The field was applied along the molecular axis *x*, see Fig. 4 of the main text for definition. The molecular geometry was optimized at every value of the field. The slope of the linear regression represents the change of polarizability upon excitation, $\Delta\alpha$.

Supplementary Figure S2. Dependence of the measured Δμ value on effective BLA parameter, calculated in Ref. 22 for the anionic GFP chromophore in 3 different proteins (full squares).

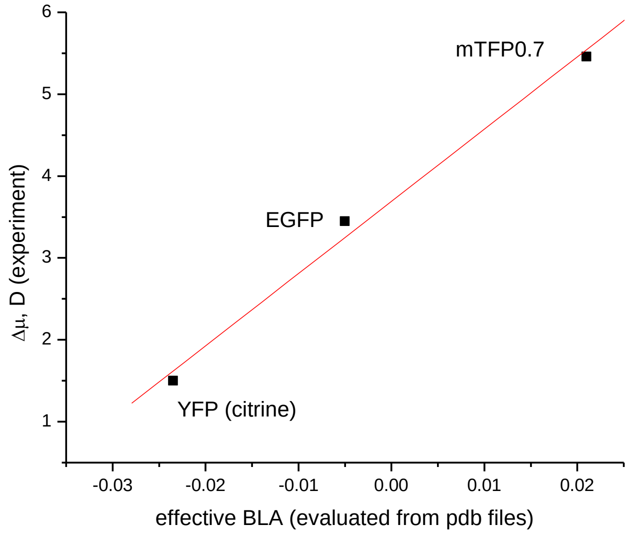

Supplementary Figure S3. Dependence of the pure electronic transition frequency on the difference between permanent dipole moments, $\Delta\mu$ for a series of GFP mutants (green circles).

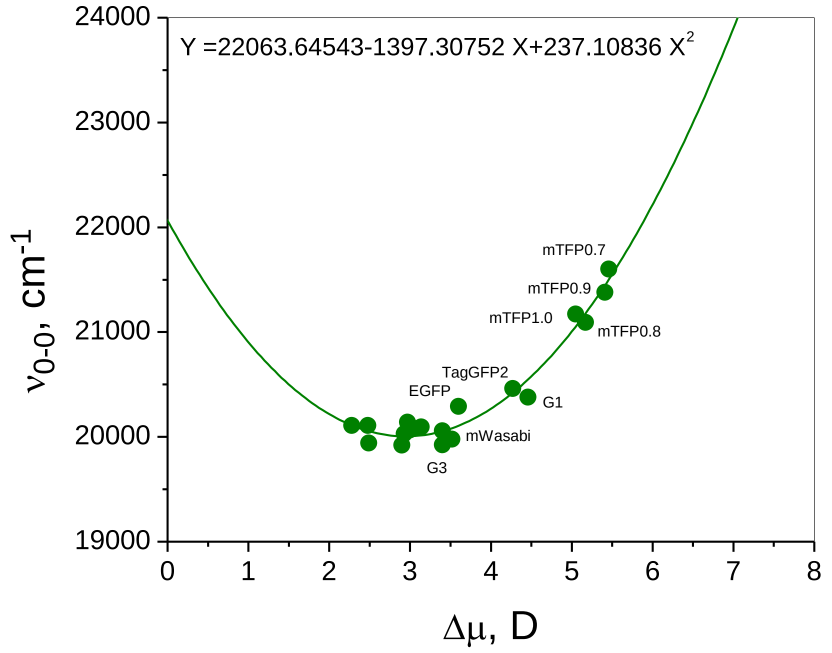

The green line is the best fit to the second-order polynomial.

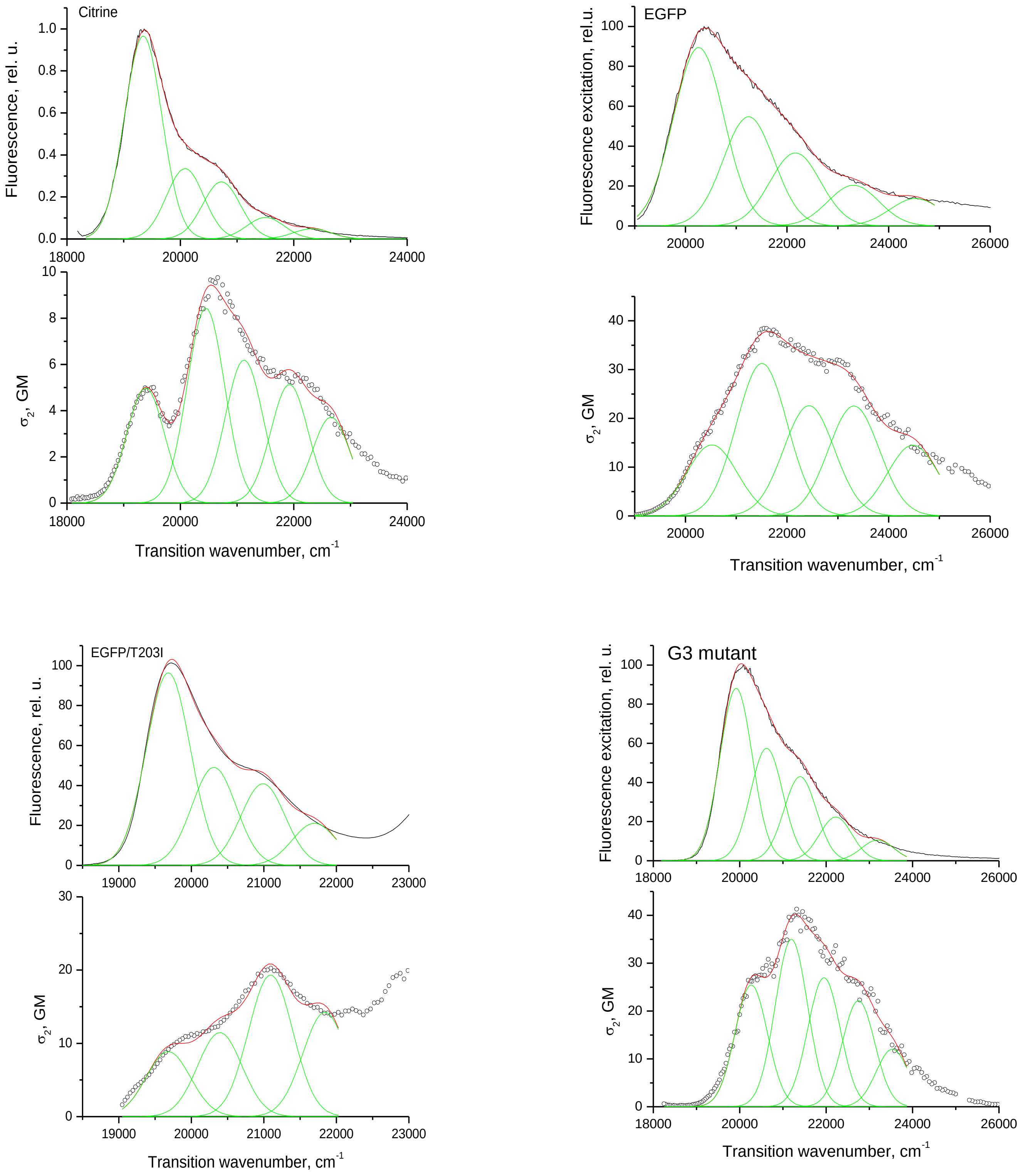

Citrine
Fluorescence, rel. u.
σ2, GM
Transition wavenumber, cm-1
EGFP
Fluorescence excitation, rel.u.
σ2, GM
Transition wavenumber, cm-1
EGFP/T203I
Fluorescence, rel. u.
σ2, GM
Transition wavenumber, cm-1
G3 mutant
Fluorescence excitation, rel. u.
σ2, GM
Transition wavenumber, cm-1

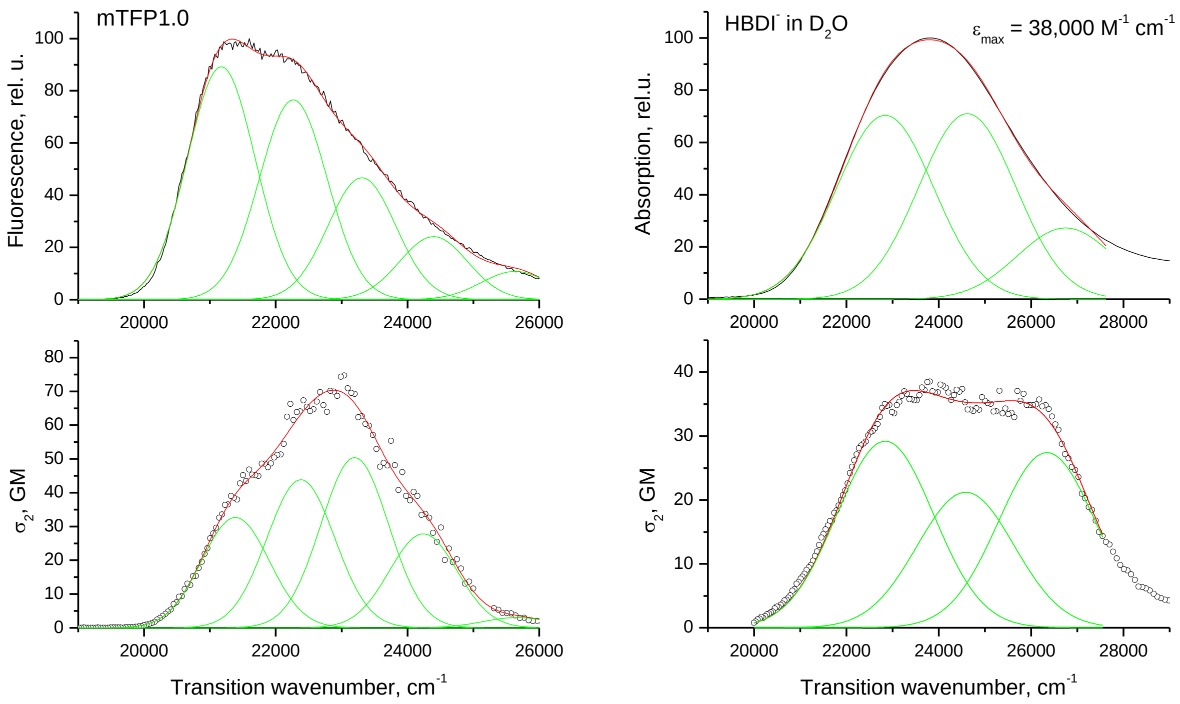

Supplementary Figure S4. One-photon fluorescence excitation spectrum (top) and two-photon excitation spectrum (bottom) with the corresponding multi-Gaussian deconvolutions for the representative series of proteins and the $HBDI^-$ chromophore in alkaline D_2O solution.

Supplementary References

1. Martin, M. E., Negri, F. & Ollivuci, M. Origin, nature, and fate of the fluorescent state of the green fluorescent protein chromophore at the CASPT2//CASSCF resolution. *J. Am. Chem. Soc.* **126**, 5452-5464 (2004).

2. Filippi C., Buda, F., Guidoni, L. & Sinicropi, A. Bathochromic shift in green fluorescent protein: A puzzle for QM/MM approaches. *J. Chem. Theory Comput*. **8**, 112-124 (2012).

3. Olsen, S. & McKenzie, R. H. Bond alternation, polarizability, and resonance detuning in methane dyes. *J. Chem. Phys*. **134**, 114520 (2011).

4. Zhang L., Xie, D. & Zeng, J., Electronic excitations of green fluorescent proteins: Modeling solvatochromic shifts of chromophore model compounds in solutions. *J. Theor. Comput. Chem*. **5**, 375-390 (2006).

5. Wanko, M., García-Risueño, P., Rubio, A. Excited states of the green fluorescent protein chromophore: Performance of ab initio and semiempirical methods. *Phys. Stat. Sol. B* **249**, 392-400 (2012).

6. Das, A., Hasegawa, J.-Y., Miyahara, T., Ehara, M., Nakatsuji, H. Electronic excitations of the green fluorescent protein chromophore in its protonation states: SAC/SAC-CI study. *J. Comput. Chem.* **24**, 1421-1431 (2003).

7. Altoe, P., Bernardi, F., Garavelli, M., Orlandi, G. & Negri, F. Solvent effects on the vibrational activity and photodynamics of the green fluorescent protein chromophore: A quantum-chemical study. *J. Am. Chem. Soc*. **127**, 3952-3963 (2005).

8. Xie, D. & Zeng, J. Electronic excitations of green fluorescent proteins: Protonation states of chromophore model compound in solutions. *J. Comput. Chem*. **26**, 1487-1496 (2005).

9. Wan, S., et al. Photoabsorption of green and red fluorescent protein chromophore anions *in vacuo*. *Biophys. Chem.* **129**, 218-223 (2007).

10. Nifosì, R., Amat, P. & Tozzini V. Variation of spectral, structural, and vibrational properties within the intrinsically fluorescent proteins family: A density functional study. *J. Comput. Chem.* **28**, 2366-2377 (2007).

11. Epifanovsky, E., Polyakov, I., Grigorenko, B., Nemukhin, A. & Krylov A. I. Quantum chemical benchmark studies of the electronic properties of the green fluorescent protein chromophore. 1. Electronically excited and ionized states of the anionic chromophore in the gas phase. *J. Chem. Theor. Comput*. **5**, 1895-1906 (2009).

12. Weber, W., Helms, V., McCammon, J. A., Langhoff, P. W., Shedding light on the dark and weakly fluorescent states of green fluorescent proteins. *Proc. Natl. Acad. Sci. USA* **96**, 6177-6182 (1999).

13. Sinicropi, A., Andruniow, T., Ferré, N., Basosi, R. & Olivucci, M. Properties of the emitting state of the green fluorescent protein resolved at the CASPT2//CASSCF/CHARMM level. *J. Am. Chem. Soc*. **127**, 11534-11535 (2005).

14. Bublitz, G. King, B. A., & Boxer, S. G. Electronic structure of the chrophore in green fluorescent protein (GFP). *J. Am. Chem. Soc.* **120**, 9370-9371 (1998).

15. Fried, S. D., Wang, L.-P. Boxer, S. G., Ren, P. & Pande V. S. Calculations of the electric fields in liquid solutions. *J. Phys. Chem. B*, **117**, 16236-16248 (2013).

16. Marder, S. R., et al. A unified description of linear and nonlinear polarization in organic polymethine dyes. *Science*, **265**, 632-635 (1994).

17. Lu, D., Chen, G., Perry, J. W. & Goddard, W. A. III. Valence-bond charge-transfer model for nonlinear optical properties of charge-transfer organic molecules. *J. Am. Chem. Soc*. **116**, 10679-10683 (1994).

18. Barzoukas, M., Runser, C., Fort, A., Blanchard-Desce, M. A two-state description of (hyper)polarizabilities of push-pull molecules based on a two-form model. *Chem. Phys. Lett.*, **257**, 531-537 (1996).

19. Laino, T., Nifosì, R. & Tozzini, V. Relationship between structure and optical properties in green fluorescent proteins: A quantum mechanical study of the chromophore environment. *Chem. Phys.* **298**, 17-28 (2004).

20. Drobizhev M., et al. Primary Role of the Chromophore Bond Length Alternation in Reversible Photoconversion of Red Fluorescence Proteins. *Sci. Rep.* **2**, 688 (2012).

21. Drobizhev, M., Makarov, N. S., Tillo, S. E., Hughes, T. E. & Rebane A. Describing Two-Photon Absorptivity of Fluorescent Proteins with a New Vibronic Coupling Mechanism. *J. Phys. Chem. B*, **116**, 1736-1744 (2012).

22. Amat, P. & Nifosì, R. Spectral "fine" tuning in fluorescent proteins: The case of the GFP-Like chromophore in the anionic protonation state *J. Chem. Theory Comput*. **9**, 497-508 (2013).

23. Shin, Y.-G., Brunschwig, B. S., Creutz, C. & Sutin, N. Toward a quantitative understanding of dipole-moment changes in charge-transfer transitions: Electroabsorption spectroscopy of transition-metal complexes. *J. Am. Chem. Soc.* **117**, 8668-8669 (1995).

24. Grisanti, L., D'Avino, G., Painelli, A., Guasch, J., Ratera, I. & Veciana, J. Essential state models for solvatochromism in donor-acceptor molecules: The role of the bridge. *J. Phys. Chem. B* **113**, 4718-4725 (2009).

25. Atkins, P. W. & Friedman R. S. Molecular Quantum Mechanics. Oxford Univ. Press, Oxford, UK, 2010.

26. Forbes, M. W. & Jockusch, R. A. Deactivation pathways of an isolated green fluorescent protein model chromophore studied by electronic action spectroscopy. *J. Am. Chem. Soc*. **131**, 17038-17039 (2009).

27. Kurimoto, M., Subramony, P., Gurney, R. W., Lovell, S., Chmielewski & Kahr, B. Kinetic stabilization of biopolymers in single-crystal hosts: Green fluorescent protein in α-lactose monohydrate. *J. Am. Chem. Soc*. **121**, 6952-6953 (1999).

28. Kammeyer, C. W. & Whitman, D. R. Quantum mechanical calculation of molecular radii. 1. Hydrides of elements of periodic groups IV and VII. *J. Chem. Phys*., **56**, 4419-4421 (1972).

29. Toptygin, D. Effects of the solvent refractive index and its dispersion on the radiative decay rate and extinction coefficient of a fluorescent solute. *J. Fluoresc.*, **13**, 201-219 (2003).
